# Pathogenic Insights into DNA Mismatch Repair (MMR) Genes–Proteins and Microsatellite Instability: Focus on Adrenocortical Carcinoma and Beyond

**DOI:** 10.3390/diagnostics13111867

**Published:** 2023-05-26

**Authors:** Mara Carsote, Ionut Florin Turturea, Maria Roxana Turturea, Ana Valea, Claudiu Nistor, Ancuta-Augustina Gheorghisan-Galateanu

**Affiliations:** 1 Department of Endocrinology, Carol Davila University of Medicine and Pharmacy & C.I. Parhon National Institute of Endocrinology, 011461 Bucharest, Romania; carsote_m@hotmail.com; 2 Department of Orthopedics and Traumatology, Cluj Emergency County Hospital, 400347 Cluj-Napoca, Romania; 3 Department of Endocrinology, Dej City Hospital, 405200 Dej, Romania; mariaroxanaturturea@gmail.com; 4 Department of Endocrinology, Iuliu Hatieganu University of Medicine and Pharmacy & Clinical County Hospital, 400347 Cluj-Napoca, Romania; ana74us@yahoo.com; 5 Department 4-Cardio-Thoracic Pathology, Thoracic Surgery II Discipline, Carol Davila University of Medicine and Pharmacy & Thoracic Surgery Department, Dr. Carol Davila Central Emergency University Military Hospital, 050474 Bucharest, Romania; 6 Department of Molecular and Cellular Biology, and Histology, Carol Davila University of Medicine and Pharmacy & Department of Endocrinology, C.I. Parhon National Institute of Endocrinology, 011461 Bucharest, Romania; agheorghisan.a@gmail.com

**Keywords:** adrenocortical carcinoma, MMR gene, MMR protein, microsatellite instability, endocrine, Lynch syndrome, surgery

## Abstract

DNA damage repair pathways, including mismatch repair (MMR) genes, are prone to carcinoma development in certain patients. The assessment of the MMR system is widely recognized as part of strategies concerning solid tumors (defective MMR cancers), especially MMR proteins (through immunohistochemistry), and molecular assays for microsatellite instability (MSI). We aim to highlight the status of MMR genes–proteins (including MSI) in the relationship with ACC (adrenocortical carcinoma) according to current knowledge. This is a narrative review. We included PubMed-accessed, full-length English papers published between January 2012 and March 2023. We searched studies on ACC patients for whom MMR status was assessed, respectively subjects harboring *MMR* germline mutations, namely Lynch syndrome (LS), who were diagnosed with ACC. MMR system assessments in ACCs involve a low level of statistical evidence. Generally, there are two main types of endocrine insights: 1. the role of MMR status as a prognostic marker in different endocrine malignancies (including ACC)—which is the topic of the present work, and 2. establishing the indication of immune checkpoint inhibitors (ICPIs) in selective, mostly highly aggressive, non-responsive to standard care forms upon MMR evaluation (which belongs to the larger chapter of immunotherapy in ACCs). Our one-decade, sample-case study (which, to our knowledge, it is the most comprehensive of its kind) identified 11 original articles (from 1 patient to 634 subjects per study diagnosed with either ACC or LS). We identified four studies published in 2013 and 2020 and two in 2021, three cohorts and two retrospective studies (the publication from 2013 includes a retrospective and a cohort distinct section). Among these four studies, patients already confirmed to have LS (N = 643, respective 135) were found to be associated with ACC (N = 3, respective 2), resulting in a prevalence of 0.0046%, with a respective of 1.4% being confirmed (despite not having a large amount of similar data outside these two studies). Studies on ACC patients (N = 364, respective 36 pediatric individuals, and 94 subjects with ACC) showed that 13.7% had different MMR gene anomalies, with a respective of 8.57% (non-germline mutations), while 3.2% had MMR germline mutations (N = 3/94 cases). Two case series included one family, with a respective four persons with LS, and each article introduced one case with LS-ACC. Another five case reports (between 2018 and 2021) revealed an additional five subjects (one case per paper) diagnosed with LS and ACC (female to male ratio of 4 to 1; aged between 44 and 68). Interesting genetic testing involved children with *TP53*-positive ACC and further *MMR* anomalies or an *MSH2* gene-positive subject with LS with a concurrent germline *RET* mutation. The first report of LS-ACC referred for PD-1 blockade was published in 2018. Nevertheless, the use of ICPI in ACCs (as similarly seen in metastatic pheochromocytoma) is still limited. Pan-cancer and multi-omics analysis in adults with ACC, in order to classify the candidates for immunotherapy, had heterogeneous results, and integrating an MMR system in this larger and challenging picture is still an open issue. Whether individuals diagnosed with LS should undergo surveillance for ACC has not yet been proven. An assessment of tumor-related MMR/MSI status in ACC might be helpful. Further algorithms for diagnostics and therapy, also taking into consideration innovative biomarkers as MMR-MSI, are necessary.

## 1. Introduction

Aberrations in DNA damage repair pathways, including *MMR* (mismatch repair) genes involved in genome integrity, predispose individuals to carcinoma development [1,2,3]. Deficiencies in the MMR system causing hypermutability or microsatellite instability (MSI) are usually assessed when screening for Lynch Syndrome (LS), either via PCR (polymerase chain reaction) or by determining MMR proteins through immunohistochemistry (IHC) analysis provided after resection/biopsy from different tumors [2,4,5,6]. MMR gene alterations may be hereditary (as seen in LS) or somatic; MMR protein status may be deficient (thus a high MSI is identified) or intact. A positive MSI status represents a surrogate marker for tumor aggressive behavior; however, high MSI makes a tumor susceptible to an immune attack, and the patient becomes a candidate for therapy with immune checkpoint inhibitors (ICPI), a modern oncological treatment [2,4,5,6,7].

Identification, at the tumor level, of an MMR protein-deficient status/high MSI should be followed by MMR gene testing (which is the gold standard for LS diagnostics); however, this assessment may not always identify a germline mutation; on the other hand, 10% of LS-related MMR germline mutations are not associated with anomalies in MMR proteins at the IHC level [2,4,5,6,7,8,9,10]. Next-generation sequences (NGSs) represent an advanced alternative to the testing strategy, while both molecular and NGS techniques contribute to better neoplasia management in terms of screening and therapy [2,4,5,6,7].

Overall, the assessment of MMR function is widely recognized as part of strategies concerning solid tumors (so-called defective mismatch repair cancers), especially IHC for MMR proteins and molecular assays for MSI status. However, MMR genetic testing might not always be conclusive; currently, there are some limits regarding the practical applicability of MMR system evaluations [11]. 

Generally, the role of the MMR gene–protein system is well known in LS, on one hand, and it has become a value tool in the oncologic field to address a potential aggressive profile of a tumor and its suitability for ICPI, on the other hand. Concerning this second category of MMR system-based practical applications, endocrine gland-derived cancers and neuroendocrine neoplasia (NEN) represent an open issue. Gene testing in adrenocortical carcinoma (ACC) is mandatory in many cases, not only for pediatrics; this is a part of a modern approach for the optimum management of an otherwise highly aggressive malignancy. The concept of assaying MMR status is expanding, while further studies are necessary to point out the endocrine practice’s fundamental importance. In a recent paper, Lalli, E. [12] highlighted the essential role of further research regarding DNA MMR pathways due to the “high mutation burden,” which widely opens the doors to new therapeutic options, such as immunotherapy [12].

### Aim

We aim to highlight the status of MMR genes–proteins (including MSI) in relation to ACC according to current knowledge (including the integration of these data with respect to other endocrine and neuroendocrine tumors). 

## 2. Materials and Methods

**Figure 1:**
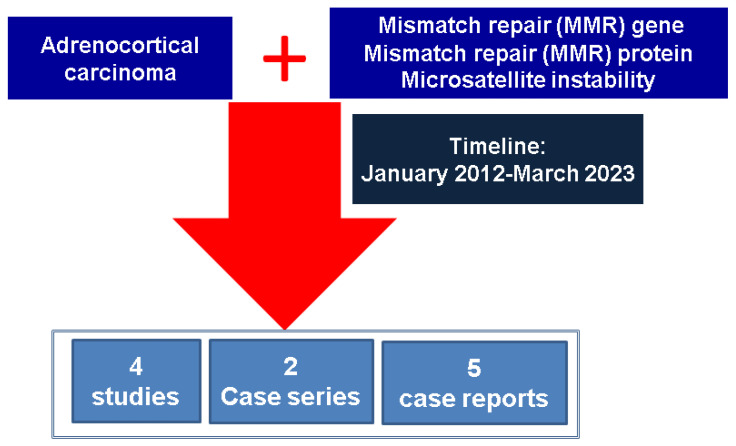
Flowchart diagram according to the methods.

This is a narrative review of the literature. We included full-length English papers published between January 2012 and March 2023 in PubMed-indexed journals focusing on MMR genes, MMR proteins, and MSI status in ACC with various levels of statistical evidence (from original studies to case reports). The research key words were, in different combinations, “adrenal” or “adrenocortical carcinoma” (alternatively, we extended the research for the Discussion section to the following: “thyroid”, “pituitary”, “endocrine”, “pheochromocytoma”, “neuroendocrine”, “parathyroid”) and “MMR” (alternatively, “mismatch repair” or “microsatellite instability” or “Lynch syndrome”). We searched studies on ACC patients for whom the MMR status was assessed either at the level of genetics, molecular testing, or immunohistochemistry, respectively, and subjects harboring *MMR* germline mutations (LS) who were diagnosed with ACC (Figure 1).

## 3. Results: MMR System and Endocrine Approach

### 3.1. DNA MMR Genes: Focus on LS

LS, an autosomal dominant hereditary syndrome, underlines mutations in four DNA mismatch repair (*MMR*) genes, *MLH1*, *MSH2*, *MSH6*, and *PMS2* [1,2,3]. Until recently, hereditary non-polyposis colorectal cancer syndrome (HNPCC) was used to describe LS, but nowadays, the term LS is accepted; clinically determined HNPCC with positive MSI includes LS (*MMR* germline mutations associating colorectal cancer (CRC) and extra-colonic tumors) and Lynch-like syndrome (negative *MMR* mutations involving CRC and rare extra-colonic cancers), while MSI-negative HNPCC is less described (involving non-*MMR* genes like *POLE* gene mutation, etc.) [3,4,5].

Found in 1 out of 250–1000 individuals, LS is considered the most frequent inherited syndrome underlying CRC and EC [4,5,6,13,14]. Other tumors are ovarian cancer (OC) and malignancies at the levels of the stomach, liver, kidney, breast, prostate, bladder, brain, and skin (Muir-Torre syndrome) [5,8,9,15].

*MLH1* and *MSH2* account for 90% of all mutations. The *MLH1* variant is associated with the highest risk of CRC, while *MSH2* mutations are associated with the most increased risk of extra-colonic neoplasia. Cancers in *MSH6* carriers occur later in life than those with *MLH1* and *MSH2*; the *PMS2* gene is related to the lowest cumulative rate of tumors; *EPCAM* mutations are more often described in relationship with CRC rather than non-colonic neoplasia [5,8,9].

The hereditary component of CRC is described in up to one third of all CRCs. LS–CRC belongs to a larger group of hereditary CRCs, including familial adenomatous polyposis, Peutz-Jeghers syndrome, Cowden syndrome, MUTYH-associated polyposis, and juvenile polyposis syndrome [8,9,16]. The life time risk of developing CRC is 80% in patients with LS, representing 3% of all cases with CRC; a surveillance protocol starts by age 20; extra-colonic tumors may be synchronous or metachronous with CRC [4,5,8,9]. EC, the second most common neoplasia in females with LS after CRC, is associated with a lifetime risk of 50%, representing 2% of all ECs; the most frequent variant is *MSH2* (cumulative incidence of 48.9% by the age of 75) followed by *MSH6*, respective of *MLH1* mutations [5,8]. 

Endocrine tumors are not part of the traditional LS spectrum; however, growing evidence shows that some tumors, such as ACC, might be associated with anomalies in the MMR system. On the other hand, MSI/MMR proteins, independently of LS, have been analyzed in relationship with certain familial non-medullary thyroid cancers (FNMTC) and NENs. 

### 3.2. ACC and MMR Status

**Figure 2:**
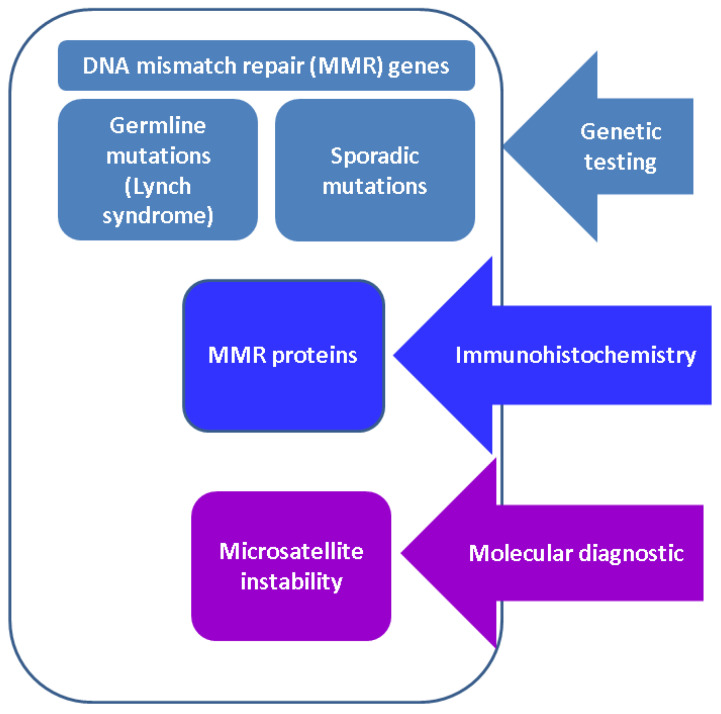
Practical approach concerning the assessment of MMR status (function).

ACC, an exceptionally rare malignancy, represents a challenging disease associated with a poor outcome. Scant data are reported in relationship with the MMR system [17] (Figure 2).

ACC has a low prevalence in the general population (between 0.5 and 2 cases/million individuals/year). Most hereditary ACC cases are in the pediatric population. Due to the substantial genetic influence, preclinical models of ACC have been developed to improve the molecular profiling of malignancy [18,19,20]. ACC is found in hereditary syndromes, such as Li-Fraumeni, familial adenomatous polyposis, and multiple endocrine neoplasia type I (MEN) [5,18,19].

ACC has been reported as a potential part of the MMR system-related spectrum, but this aspect is not entirely proven [21,22]. Moreover, MMR pathways might be contributors to ACC growth independently of LS [23]. A recent integrative pan-cancer analysis suggested that *MSH6* expression is correlated with a poor prognosis, including in ACC [24]. A study from 2021 that included 634 individuals with LS from 220 families (all were carrying an *MSH2* germline mutation) registered between 1999 and 2018 showed that the prevalence of ACC was 0.47% (3 cases), all with the loss of expression of MSH2 and MSH6 proteins [22].

One study from 2013 aimed to establish the prevalence of LS among individuals diagnosed with ACC [25]. On one hand, 82.5% of 114 patients confirmed as having ACC were tested for gene anomalies; three subjects were identified with *MMR* gene mutations, all having a family history of LS. Thus, an ACC prevalence of 3.2% was confirmed, which generally is similar to the prevalence of LS among cases with CRC or EC (ACC being considered an orphan disease due to its extremely rare incidence) [25]. On the other hand, a retrospective analysis of 135 individuals with *MMR* gene mutations who were included in the University of Michigan Cancer Genetics Registry identified two cases with ACC. IHC analysis was feasible for four tumors, all of them had MSI, and three out of four tumors had MMR protein anomalies similar to the MMR gene status [25]. 

In 2021, the first case with the co-occurrence of two mutations involving LS and MEN2A syndrome was reported in a 44-year-old adult woman confirmed as having ACC. She had a family history of LS; however, in addition to an *MSH2* gene loss of heterozygosity (LOH) (c.211+1G>T), a *RET* germline mutation (c.2410G>A; p.Val804Met) was identified. The clinical presentation was consistent with LS, but not with MEN2A syndrome. The interference between these two mutations is yet to be determined [21]. 

A retrospective study of 36 children with ACC coming from southern Brazil, where the *TP53* gene mutation (*TP53* p.Arg337His) prevalence is significantly increased, included IHC and NGS for MLH1, MSH2, MSH6, and PMS2; 8.57% of them had altered *MLH1* (N = 2 cases), respective of *MSH6* (N = 1) confirming LS, a prevalence that is higher than the prevalence of LS-associated *MMR* anomalies among global subjects with EC and CRC [26]. 

Adults with ACC are diagnosed with germline mutations in 10% of cases, the most known being Li-Fraumeni syndrome (*TP53* mutation) [27]. The first family with germline *MSH2* gene mutation-related adult ACC was published in 2016 [28]. The proband was a 54-year-old female from a family known to have LS who was diagnosed with ACC after prior therapy for CRC and ovarian malignancy; her mother died of ACC, while her sister was recognized as having CRC and EC. IHC confirmed the loss of MSH2 and MSH6 protein expression in both ACCs (also found in the EC of the sister); the deletion of exons 1–3 belonging to the *MSH2* gene was confirmed in the proband and her sister [28]. 

In females with LS, abnormal uterine bleeding (mostly post-menopausal) requires a differential diagnosis of EC with an estrogen-producing ACC. A 65-year-old women coming from a family with LS (*MSH6* mutation) was reported with post-menopausal metrorrhagia due to ACC as the first manifestation of LS. At some point, ACC may be the only tumor of a patient with LS, but this is still based on poor statistical evidence [29]. Moreover, we mention a 68-year-old male with LS who was diagnosed with ectopic (retroperitoneal) ACC harboring an *MSH2* mutation with a negative blood hormonal profile with respect to the adrenal cortex [30]. 

Gene testing in ACCs is important, including in adult cases; this is part of a modern approach to providing optimum management for an otherwise extremely aggressive malignancy [25,28]. The MMR/MSI configuration might prove useful if IHC is feasible after adrenalectomy or metastasis resection [5,25,28]. MMR aberrations serve as a novel surrogate biomarker regarding the potential response to ICPI, which does not represent the standard care in ACC [7,31,32,33]. Cancers underlying LS are particularly sensitive to ICPIs, such as pembrolizumab; we do not have enough statistical evidence to sustain the same results for ACC, but since ACC prognosis is severe, ICPI should be initiated in selected cases [33,34]. The first case of LS-ACC treated with PD-1 blockade was published in 2018 in a 58-year-old female admitted for (adrenal) Cushing’s syndrome with rapid tumor progression and a poor outcome [34]. 

The importance of MMR anomaly identification was also highlighted by a large cohort study published in 2021, which reported the highest prevalence of *MMR* mutations among patients diagnosed with ACC. Among 364 individuals with ACC (median age of 52 years), 15 subjects had some of 29 types of *MMR* genomic alterations, making them candidates to ICPI [35]. So far, we cannot confirm that individuals diagnosed with LS should undergo surveillance for ACC [5,27,29]. No specific endocrine case-finding protocol has been generally implemented up until the present time for LS (other than surgical menopause for gynecologic cancers) [5,29]. However, MMR proteins/MSI might prove helpful in selected cases when traditional management lines are inefficient. Another particular aspect includes the potential interaction of mitotane with the *MMR* status. In addition to ionizing radiation, ACC cell lines treated with mitotane are prone to cell death through a DNA damage repair system [36]. 

## 4. Discussion

**Figure 3:**
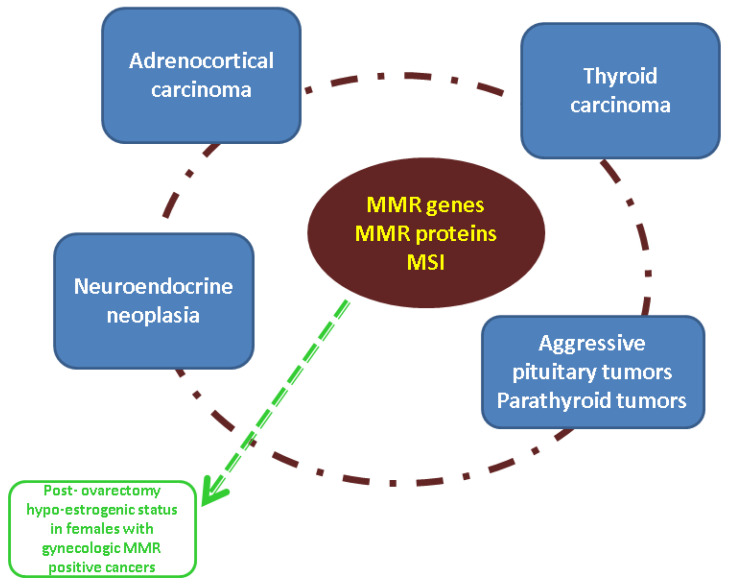
Qualitative analysis of potential endocrine issues in patients with anomalies of MMR genes, MMR proteins, or MSI status (please see selective references [17,18,19,20,21,22,23,24,25,26,27,28,29,30,31,32,33,34,35,36,37,38,39,40,41,42,43,44,45,46,47,48,49,50,51,52,53,54,55,56,57,58,59,60,61,62,63,64,65,66,67,68,69,70,71,72,73,74,75,76,77,78,79,80,81,82,83,84,85,86,87,88,89,90,91,92,93,94,95,96,97] according to main text).

Despite reduced information in the area of MMR status and ACC [21,22,25,26,28,29,30,34,35], recent data pointed out that the MMR system should be taken into consideration in other endocrine domains, particularly thyroid cancer (TC) and NENs and, from our point of view, the data on MMR-ACC should be discussed, in addition to the broader spectrum of MMR-associated endocrine tumors (Figure 3).

### 4.1. TC and MMR/MSI Involvement

The topic concerning MMR/MSI status of TCs is relatively new. The distinction between the influence of LS on thyroid cancer or MMR/MSI anomalies in subjects with TC is mandatory. However, the current level of understanding is based on isolated studies [37,38,39,40,41,42,43,44,45,46,47,48,49,50,51,52,53,54]. TC, the most frequent endocrine cancer, is associated with increasing incidence, and some particular forms have high genetic susceptibility [38]. A thyroid nodule on a patient with an *MMR* germline mutation may be accidental due to the high prevalence of thyroid nodules in the general population, an unusual site of metastasis from LS-associated malignancies (although the thyroid is a not a typical site of metastasis) [39,40,55]. A sporadic TC in a patient with LS might be related to non-*MMR* gene mutations, such as *BRAF, RAS, TP53*, etc. [41,42,56]. A case-control study on MMR gene SNPs (single nucleotide polymorphisms) concerning 106 Caucasian subjects diagnosed with well differentiated TC versus 212 (age and gender matched) controls analyzed the genetic susceptibility of six MMR SNPs (single or in combination), namely MLH1 rs1799977, MSH3 rs26279, MSH4 rs5745325, PMS1 rs5742933, MLH3 rs1750, and MSH6 rs1042821, which was associated with the follicular histological subtype of thyroid neoplasia and female sex, while in combination with the other two MMR SNPs, an MSH6 heterozygous genotype was correlated with a tumor risk reduction, confirming the potential role of MMR SNPs in differentiated carcinoma susceptibility [38].

MLH1, MLH3, PMS2, and PMS1 proteins are enzymes required to assist in the normal genomic activity of follicular thyroid cells [43]. One small sample-size, experimental study suggested a different intervention for papillary versus the follicular histological type of differentiated TC. All four MMR proteins were reduced in the papillary TC (N = 18) versus para-carcinoma normal thyroid (N = 9), also associating with reduced expression of the FOXO transcription factor as a potential promotor of PMS2 suppression. At the same time, follicular carcinoma and adenomas displayed normal MMR activity, suggesting a non-MMR-related pathway [43]. Whether MMR system intervention in different histological subtypes of non-medullary thyroid neoplasia is distinct is still an open issue. 

The modern concept of FNMTC, a particular TC subgroup with an. autosomal dominant inheritance, is related to limited data on the MMR system [37,44]. The co-occurrence of FNMTC and HNPCC may be part of LS; the presence of a TC in a patient with CRC represents an indication for checking the MMR/MSI status [37,45]. A study from 2021 found MSI in 7.4% of 175 cases with anaplastic TC, *MSH2* mutation (33%), *MSH6* mutation (25%), *MLH1* gene mutation (16.7%), and two mutations as follows: *MSH2–MSH6* (8.3%), *MLH1–MSH2* (8.3%), *MLH3–MSH5* (8.3%), and no mutation at *PMS2* (somatic, no hereditary involvement) [46]. A cohort of 28 anaplastic TCs found that 14% had MMR protein deficiency, 4/4 with MSH2 and MSH6 loss and no involvement of MLH1 and PMS2 [48]. Another study on the molecular association between FNMTC and HNPCC was performed on 43 families with FNMTC (383 participants). WES (high-throughput whole-exome sequencing) was performed on the peripheral blood DNA of 168 participants (54 of them with FNMTC). One family was identified with FNMTC and HNPCC, and a heterozygous missense variant of the *MSH2* gene (rs373226409; c.2120G>A; p.Cys707Tyr) was confirmed, but not considered an etiological factor. FNMTC-based IHC reported negative results for MSH2 and MSI, while the underlying mutation remained unknown; further considerations of the other *MMR* genes are mandatory [37]. MSI was found in 2.5% of sporadic follicular TCs. This is the result of a large cohort study that included 485 TCs; only the follicular group had a high-MSI status (N = 4/156), in addition to MMR protein deficiency based on IHC; WES was used in 2/4 cases and one had an MSH2 mutation [46]. A cases series on FNMTC-HNPCC (N = 4) in LS showed two cases with papillary TC, respectively, a 47-year-old female with a history of HNPCC, EC, and OC carrying a germline MLH1 mutation (1858G>T/E620X) and a 34-year-old male with HNPCC carrying an MSH2 mutation; one case was a 39-year-old female with an anaplastic type and HNPCC, and one case was a 44-year woman with undifferentiated TC and HNPCC [49].

An assessment of MSI/MMR status may serve as a prognostic marker in endocrine neoplasia; potentially, it brings some value in understanding the response to radioiodine therapy in a subgroup of well differentiated TCs [47,48]. For instance, a retrospective study published in 2022 on 241 individuals with TC examined the MMR protein status (either deficient or intact): 7.5% of TCs (N = 18 cases, as follows: N1 = 12 papillary TCs, N2 = 2 poorly differentiated TC, N3 = 4 anaplastic TCs) were MMR-deficient for all four MMR proteins, which correlated with the fact that 50% of them had an MSH6 deletion, while 50% of them underlined variants of MSH6 and PMS2. Interestingly, 22% of N was PD-L1-positive, which is associated with a shorter recorded overall survival versus MMR-deficient-PD-L1 negative subjects [57]. This study emphasizes the importance of the MMR status in TC in terms of prognosis and options for therapy, which is otherwise not routinely necessary since most differentiated TCs have an excellent prognosis. Further models of prediction and protocols to implement ICPI among the subgroups with aggressive TCs are necessary to expand the MMR concept in endocrine tumors. 

Malignancies synchronous to TC might be a clue of a hereditary syndrome [45,50]. In 2018, the first patient with LS with multiple, simultaneous head and neck cancers was diagnosed: papillary TC, adenocarcinoma at the level of the salivary gland, and acinic cell carcinoma [51]. A study based on 368 carriers from 176 families with LS identified 46.8% of them with CRC (especially MLH1 carriers) and 53.2% (N = 268) with non-colonic cancers (especially MSH2, MSH6, and PMS2 carriers); three cases out of 268 with extra-colonic cancer had a TC, which at that point (in 2012), was not considered part of LS [52].

The role of molecular and genetic testing (including MMR status) as part of a modern approach of TC is continuously increasing, as similarly seen in ACC. Of course, the epidemiologic impact of the two malignancies is distinct, and this might explain why limited data are published on ACC versus TCs. The assessment of MMR/MSI seems to add an interesting and valuable piece to this challenging puzzle of these endocrine cancers [42,48,53,54].

For practical purposes, we mention the first case of a benign multi-cystic thyroid disease with hemorrhagic transformation in a 45-year-old female with LS without other neoplasia who was under a cancer surveillance protocol [58]. This is a potential complication of a pharyngeal intubation, knowing that individuals with LS need systematic procedures for neoplasia screening and follow-up; moreover, the use of anticoagulant/antiplatelet medication might be an aggravating factor under these specific circumstances [58].

### 4.2. NEN and MMR/MSI Assessment

Recently, it was suggested that NEN is part of the extra-colonic tumor presentation in LS; however, not all authors agree [59,60]. Most data on LS-NEN are reported for the gastrointestinal site, an area that is intensively explored due to the high prevalence of CRC [61,62,63]. One study based on upper gastrointestinal findings in 323 asymptomatic patients diagnosed with LS (median age of 49.5 years at baseline) identified, via 717 eso-gastro-duodenoscopies (each individual had at least two procedures performed after 2.3 years), the following: 1.5% of them (N = 5) had early-stage cancers, and one of them was a gastric NEN [64]. Regarding lower gastrointestinal NENs, we mention a case series of six patients with germline mutations underlying colorectal NENs; 3/6 subjects had LS (1/3 with well-differentiated NEN, and two cases with neuroendocrine carcinoma) [65]. In 2022, a 49-year-old female was identified carrying an *MSH2* missense mutation c.1808A>T, while she successively had EC, lymphoma, colonic cancer, gastric adenocarcinoma, and neuroendocrine carcinoma (IHC on NEN showed reduced expression of the MSH2 protein) [66]. 

The coexistence of adenocarcinoma and NEN at the same gastrointestinal tumor was reported early in LS [67]. For instance, we mention a 63-year-old female with an initial diagnosis of adenocarcinoma of the colon and stomach, which was diagnosed with liver metastasis of high-grade NEN originating from a gastric remnant; she had an LS-germline *MLH1* mutation, in addition to the loss of MLH1 protein in NEN and adenocarcinoma of the stomach [67]. Lately, the concept of mixed neuroendocrine non-neuroendocrine neoplasms (MiNENs) was extensively developed; MiNENs are mostly gastro-entero-pancreatic NEN with the very aggressive behavior of both NEN and non-NEN [68]. A study on 44 patients with MiNEN (mean age of 61 years; 63.6% gastric MiNEN) explored IHC for MLH1, MSH2, MSH6, and PMS2: a lack of MMR protein expression was identified in 38.6% of them (most frequent: 29.4% for MLH1/PMS2, respective of 23.5% for MLH1); MMR deficiency versus normal MMR status correlated with a better prognosis in MiNEN [68].

Some data involve pancreatic NEN in LS, while pancreas cancer is part of LS-related cancers, mostly involving the adenocarcinoma type with a distinct feature due to a particular ICPI response [69,70,71]. Other hereditary syndromes also underlying pancreas malignancy and hormonally active tumors include von Hipple-Lindau disease, McCune-Albright, Li-Fraumeni, MEN1, and Beckwith-Wiedemann syndrome [72,73]. A case series published in 2012 included the first report of a patient with LS associating a pancreatic, well-differentiated neuroendocrine tumor [74]. However, only in 2017, was the first case of pancreatic NEN and IHC confirmation in LS was reported: a 65-year female with LS and a history of two CRCs, EC, and ductal mammary neoplasia was diagnosed with non-functioning pancreatic NEN (loss of expression of MLH1 and PMS2 was identified via IHC and MSI through PCR) [75]. A recent study on pancreatic NENs and IHC for all four MMR proteins found no correlation with tumor progression [76]. The major implications of the MMR system in NENs relates to the potential response to immunotherapy [77]. 

Additionally, the broader NEN spectrum includes the neuroendocrine component of gynecological cancers displaying anomalies of MMR genes–proteins [62,63]. A single-center study of 104 cases of EC (median age of 53 years) identified 48% (N = 50) with an MMR-deficient status via IHC (N = 33/50: loss of MLH1 and PMS2, N = 14/50: loss of MSH2 and MSH6, N = 2/50: loss of MSH2, N = 1/50 loss of PMS2) and 2/50 of these had focal neuroendocrine differentiation [78]. The first case of NEN at the uterine cervix was reported in 2014 based on a 34-year-old female from a family with LS; the tumor was the first manifestation of the syndrome; small cell neuroendocrine carcinoma was diagnosed; IHC confirmed the loss of MLH1 protein expression [79]. 

We identified a single paper (2020) referring to pheochromocytoma in a 33-year-old patient with LS [59]. Interestingly, another report introduced a 57-year-old female with LS first diagnosed with pheochromocytoma by the age of 51. Lately, it was confirmed to be a metastatic ACC. In addition to surgery, mitotane and immunotherapy with pembrolizumab were added, a notably important line of therapy in LS-associated endocrine neoplasia [80]. In 2023, a large study on metastatic paraganglioma-pheochromocytoma syndrome showed that MSI is among the elements associated with ATRX/TERT alterations, having value as a prognostic marker [81].

Currently, MSI and MMR protein deficiency have been explored in different malignancies, particularly to address an indication of ICPI [82,83,84,85,86,87,88]. Exploring the MMR/MSI status should become the new standard in poorly differentiated NEN, regardless of germline mutations consistent with LS [85,86,87,88].

### 4.3. Integrating MMR Assessment into the Larger Panel of Endocrine Tumors 

Concerning pituitary tumors, limited data have been published so far; we identified five relevant papers, the majority of them highlighting an unusually aggressive behavior [89,90,91,92,93]. Resistance to standard therapy indicates PD-1 blockade if MMR anomalies are confirmed, despite insufficient statistical evidence concerning the pituitary field [89,90,91]. Deficiencies of MSH2 and MSH6 proteins are linked to pituitary adenoma proliferation [94]. In 2021, a 56-year-old women was reported with an *MSH2* mutation and aggressive prolactinoma, and another carrying the same mutation was diagnosed with an invasive corticotropinoma [90,93]. Another unusual case of a 56-year-old women with LS harboring both *MSH2* and *MSH6* mutations included the onset of the pituitary tumor with apoplexy, and panhypopituitarism was followed by self-remission, and two years later, recurrence was confirmed with the identification of an undifferentiated pituitary carcinoma with pulmonary and osseous spreading [91]. A first case of pituitary adenoma and LS was published in 2017 based on a 68-year-old subject with LS who was recognized as having a rapidly progressive corticotropinoma; she had an *MLH1* germline mutation and an *MSH6* somatic mutation, but also an *MEN1* somatic mutation; IHC was negative for MLH1 and MSH6 [92]. Also published in 2017, we identified a single study on pituitary tumors in subjects with LS; this was a nationwide study comprising 910 individuals with LS (Swedish National Patient Registry) that identified three cases with pituitary tumors: a prolactin-secretor microadenoma, an invasive non-secreting pituitary macroadenoma, and another case with a very unusual presentation, a locally aggressive corticotropinoma causing a severe form of endogenous Cushing’s syndrome in a 51-year-old male, which was recognized as pituitary carcinoma due to hepatic metastasis after 16 months since the first diagnosis [89]. The authors of this study suggest the importance of establishing the underlying *MMR* gene mutations in individuals with aggressive pituitary tumors since they may be subject to ICPI therapy, mainly seen with other LS-related tumors [89]. 

Of note, two papers (one from 2020 and another from 2012) reported a parathyroid tumor in a patient with LS [95,96]. One case was a 65-year-old lady confirmed as having parathyroid carcinoma–related primary hyperparathyroidism; the other case introduced a subject with LS underlying an *MLH1* gene mutation and a missense mutation of the *APC* (adenomatous polyposis coli) gene [95,96]. We do not have enough evidence to include parathyroid tumors among the LS panel of tumors and do not routinely recommend the assessment of MSI/MMR proteins in parathyroid carcinoma.

### 4.4. Endocrine Considerations in Patients with Germline MMR Mutations

We consider it useful to briefly integrate the role of the endocrinologist in the management of patients harboring germline *MMR* mutations, namely a multidisciplinary surveillance team to address the reproductive endocrinology issues in LS females diagnosed with gynecological cancers or referred for prophylactic gonadectomy while being *MMR* positive. EC is the second most frequent malignancy in LS, and OC is less often reported; breast malignancy is sometimes associated with the syndrome, but not all authors agree; on the other hand, some ECs (outside LS) display anomalies of MMR proteins without germline mutations [5,8,97]. The highest rate of mutations in EC involves the *MSH2* gene; carriers of *MLH1* mutations are associated with a cumulative incidence of 37% by the age of 75; *MSH6* anomalies have a higher risk of EC than other non-CRCs; for *PMS2* variants, the presentation is later in life, while the risk is the lowest compare to that with other *MMR* genes [5,8]. Periodic check-up is recommended by the age of 30; in some females, the risk of EC is even higher than CRC depending on the MMR gene/protein status; 60% of females with LS have EC as the first malignancy [5,8,98]. Clinical characteristics of LS-EC are young adults, a lower body mass index, and endometrioid histological type [99]. Prophylactic hysterectomy and bilateral salpingo-oophorectomy are recommended depending on the women’s desire to have children, while menopause and females who require surgery for extensive CRC are more often taken into consideration [5,98,99,100]. Pembrolizumab and nivolumab may be recommended for an abnormal MSI/MMR status in EC [5,98,99,100]. 

OC correlates with *MLH1*, *MSH2*, and *MSH6* mutations (the most frequent is *MSH2* with a cumulative rate of 10–17% by the age of 75); the onset is typically after age 40; no specific guidelines address the issue of fertility preservation in LS-OC [5,8,101]. Breast cancer (BC) has a cumulative incidence of 12–17% by age 75, depending on the pathogenic variant [5,8]. One study found that the *PMS2* mutation is associated with the highest risk among four *MMR* genes (mean age at diagnostic of 46.7 years) [102]. The *MMR* gene–protein system has been studied in hormone-receptor-positive BC, especially those with a poor response to standard management. The identification of *MMR* mutations helps in the early detection of BC in hereditary circumstances, such as LS. Approximately 3% of BCs with standard therapy resistance have different types of MMR anomalies [103]. 

Menopause-related symptoms are expected after gonadectomy or medically induced hypogonadism in females with LS [5,103,104,105]. Prophylactic gynecological surgery negatively impacts females’ quality of life, except for the reduced worry related to the further development of EC or OC [106]. Surgically induced menopause might be associated with a more severe presentation than spontaneous menopause [104]; in the absence of hormone therapy, vasomotor and local vaginal symptoms, in addition to long-term complications, might be expected in terms of osteoporotic fracture risk and cardiovascular and cognitive impairment [103]. Growing evidence shows the importance of actively pursuing gene testing, especially for *BRCA1/2* and *MMR* genes, in women diagnosed with gynecological malignancies in cases without a clear familial diagnosis, especially at younger ages and in cases already diagnosed as CRC [103]. Pregnancy issues in females with LS include genetic counseling (there is a 50% chance of inheriting the pathogenic mutation); LS-associated cancer screening should be performed before pregnancy; if a new tumor is diagnosed during pregnancy, the management is individual [5,107]. CRC reduces the fertility rate in females, but not males [103]. *MMR* genes are related to meiotic recombination during spermatogenesis, and infertile men might experience *MMR* gene anomalies, independent of LS [108,109]. Concerning male gonads, testicular tumors are not part of the LS configuration; however, it was speculated that aggressive testicular germ cell tumors with resistance to chemotherapy might be associated with MMR/MSI anomalies, an aspect that is still under debate [110,111]. 

### 4.5. Current Data on the MMR-MSI System in ACCs: Is There a Next Step?

*MMR* genes, but mostly MMR proteins and MSI assays, represent a modern topic in approaching endocrine and neuroendocrine tumors. Moreover, the patients diagnosed with *MMR* germline mutations according to LS display various endocrine issues, as mentioned. Overall, the MMR system and its assessment in ACCs currently involve a low level of statistical evidence [21,22,25,26,28,29,30,34,35,74,80]. We anticipate an extension of these data as recently seen in the area of TCs and NENs.

Generally, there are two main types of endocrine insights: the role of MMR status as a prognostic marker in different endocrine malignancies (including ACC) on one hand (which is the topic is the present work), and, respectively, establishing the indication of ICPI in selective (mostly highly aggressive, non-responsive to standard care) forms upon MMR evaluation (which belongs to the larger chapter of immunotherapy in ACCs). Our one-decade-based sample-case study identified a number of 11 original articles (from 1 patient to 634 subjects per study diagnosed with either ACC or LS). We looked for studies on LS patients seeking an ACC identification (underlying germline *MMR* mutations), as well as studies of MMR genes and proteins or MSI assays in subjects confirmed as having ACCs [21,22,25,26,28,29,30,34,35,74,80] (Table 1).

We identified four studies that were published in 2013 and 2020 and two in 2021, three cohorts and two retrospective studies (the publication from 2013 includes a retrospective and a cohort distinct section) [22,25,26,35]. Among these four studies, patients already confirmed as having LS (N = 643, respective 135) were found to be associated with ACC (N = 3, respective 2 cases), thus a prevalence of 0.0046% was obtained, respective of 1.4% being confirmed (despite not having a large amount of similar data outside these two studies) [22,25]. In contrary, studies on ACC patients (N = 364, respective 36 pediatric individuals, and 94 subjects with ACC) showed the following MMR anomalies: 13.7% had different MMR gene anomalies, respective of 8.57% (non-germline mutations), while 3.2% had LS (MMR germline mutations, N = 3/94 cases) [25,26,35]. Two case series (from 2012 and 2016) included one family, respective of four persons with LS, and each article introduced one case with LS-ACC [28,74]. Another five case reports (published between 2018 and 2021) revealed an additional five subjects (one case per paper) diagnosed with LS and ACC (female to male ratio of 4 to 1; aged between 44 and 68 years) [21,29,30,34,80]. An interesting genetic diagnosis involved children with *TP53*-positive ACC and further *MMR* anomalies or an *MSH2* gene (c.211+1G>T)-positive subject with LS with a concurrent germline *RET* mutation (c.2410G>A; p.Val804Met) [21,26] (Table 2).

The first report of LS-ACC referred for PD-1 blockade was published in 2018 [34]. Nevertheless, the use of ICPI in ACCs (as similarly seen in metastatic pheochromocytoma) is still limited. One aspect is caused by the need for particular molecular and immunohistochemistry diagnostics (including MMR assays, but many other pathways are reported) in order to indicate the suitable candidates. Perhaps this class better improves the outcome in association with mitotane, tyrosine kinase inhibitors, or traditional chemotherapeutics rather than a single line of medical management, an aspect that is yet to be determined [112,113]. For instance, a single-center experience with pembrolizumab in terms of a phase II clinical trial was based on 16 subjects with ACC (50% females; 63% with hormonally active profile), and 93% of them were microsatellite stable (1/16 with positive MSI); the drug showed only a modest benefit regardless the MSI status [114]. A pan-cancer and multi-omics analysis in adults with ACC, in order to classify the patients for immunotherapy, has had heterogeneous results, and the integration of the MMR system in this larger and challenging picture is still an open issue [115,116,117]. 

The evaluation of MMR/MSI status as part of the current assessment for both pediatric and adult patients with ACC represents one step to the future. Currently, we may only assume that by testing this system in terms of genetic, molecular, and immunohistochemistry analysis, we might improve our understanding of multiple pathogenic mechanisms underlying this aggressive malignancy and additionally be able to initiate an alternative line of medication, such as, for instance, ICPI. In the modern era, ACC management in terms of diagnosis includes a complex hormonal, imaging, and histological/molecular workout. The scenario of detection varies from endocrine anomalies caused by adrenal hormones, such as high blood pressure, adrenal Cushing’s syndrome, and interference of puberty in children, to the incidental detection of a large adrenal mass (adrenal incidentaloma), typically larger than 4–10 cm, upon various investigations, such as abdominal ultrasound, computed tomography, magnetic resonance imaging, etc. Gene testing is mostly used in children and teenagers. Post-operatory findings (regardless of whether the tumor has been entirely removed) provide confirmation of the histological report and immunohistochemistry features that might serve as prognostic markers, such as Ki67, Weiss, and Wieneke scores, etc., with none of these being able to provide a 100% accurate prediction. The Weiss score seems to be less useful in children. The prognosis remains poor, with surgery in addition to mitotane, as well as chemotherapy in selected cases, being able to provide a reduced 5-year survival [118,119,120,121]. Whether a subgroup of these patients are at a higher risk by showing MMR/MSI anomalies or whether we should routinely check MMR status in order to decide on a specific adjuvant therapy is still a matter of debate.

## 5. Conclusions

Whether individuals diagnosed with LS should undergo surveillance for ACC is still an open issue, and so far, it is not regarded as part of LS. The MMR system’s role in endocrine tumorigenesis is yet to be determinate. Practitioners should be aware of this pathogenic loop. So far, we have data on thyroid malignancies and neuroendocrine neoplasia, in addition to promising results for ACC. The data on this particular matter of an adrenal cortex-derived malignancy should be cautiously applied, noting that, on one hand, the tumor is very aggressive, while, on the other hand, this type of cancer is very rare, and thus, the typical level of statistical evidence has hardly been achieved in most directions of diagnosis and therapy. Of note, the use of ICPI is rather recent, representing one of the most dynamic chapters in oncologic endocrinology. Exploring the tumor-related MMR/MSI status in endocrine tumors might provide a valuable prognostic marker and help in the selection of ICPI candidates. Further evaluation algorithms, prediction models, and associated management decisions are necessary.

## Figures and Tables

**Table 1:** The spectrum of studies on ACC in relationship with MMR genes–proteins and MSI status (the display is based on statistical significance: from studies to case reports starting with the most recent) [21,22,25,26,28,29,30,34,35,74,80].

First Author Reference	Year of Publication	Type of Study	Participants	MMR Gene, MMR Proteins, and Microsatellite Instability Status
**Studies**				
Domènech. M. [22]	2021	Cohort study	634 individuals with LS coming from 220 families	0.47% prevalence of ACC (N = 3) All individuals carried a *MSH2* germline mutation 3 subjects with ACC: loss of expression of MSH2 and MSH6 proteins
Pozdeyev, N. [35]	2021	Cohort study	364 individuals with ACC (median age of 52 y)	13.7% of them with *MMR* genes mutations
Brondani, V.B. [26]	2020	Retrospective study	36 children with ACC caring *TP53* p.Arg337His germline mutation (southern Brazil)	3 cases (8.57%) with *MMR* anomalies: 2 patients with *MLH1* mutation 1 patient with *MSH6* mutation
Raymond, V.M. [25]	2013	Cohort study Retrospective study	94 patients with ACC (prospective gene counseling) 135 individuals with LS (University of Michigan Cancer Genetics Registry) (retrospective analysis)	3.2% prevalence of LS (3 cases) 2 cases with ACC IHC available for 4 ACC: 4/4—MSI 3/4—similar MMR protein-gene configuration The prevalence of LS among patients with ACC is similar with LS prevalence among patients with CRC and EC
**Case series**				
Challis, B.G. [28]	2016	Case series	1 family with LS	One family with adult ACC Proband: 1 female (54 y) with ACC (prior diagnostic of colorectal cancer and ovarian cancer) Her mother: ACC with fatal outcome Her sister: colorectal cancer and EC
Karamurzin, Y. [74]	2012	Case series	4 cases with LS	1 case with ACC (1 case with pancreatic well-differentiated NET)
**Case reports**				
Raygada, M. [21]	2021	Case report	1 female with LS (44 y)	ACC Loss of heterozygosity of *MSH2* gene (c.211+1G>T) This is the first case with a second germline mutation: *RET* gene mutation (c.2410G>A; p.Val804Met), without clinical manifestation of MEN2A syndrome
Shetty, I. [80]	2020	Case report	1 female case with LS (57 y)	Diagnosis of ACC at 51 y Pathogenic mutation (p.Q46X) in *MSH2* gene IHC: loss of expression for MSH2 and MSH6 proteins
Kaur, R.J. [29]	2019	Case report	1 female case with LS (65 y)	Diagnosis of ACC at 65 y *MSH6* mutation
Casey, R.T. [34]	2018	Case report	1 female case with LS (58 y)	Diagnosis of ACC at 58 y Pathogenic mutation in *MSH2* (p.Asn263fs) This the first case of LS-ACC treated with PD-1 blockade
Wright, J.P. [30]	2018	Case report	1 male case with LS (68 y)	Diagnosis of ACC at 68 y *MSH2* mutation Ectopic presentation and hormonally inactive ACC

Abbreviations: ACC = adrenocortical carcinoma; CRC = colorectal cancer; EC = endometrial cancer; IHC = immunohistochemistry; LS = Lynch syndrome; MMR = mismatch repair genes; MSI = microsatellite instability; NET = neuroendocrine tumor.

**Table 2:** Overview of sample case-based analysis of ACC-MMR/MSI [21,22,25,26,28,29,30,34,35,74,80].

Parameter	Outcome
level of evidence ACC-MMR/MSI	low (however, ACC is an orphan disease)
2 types of approaches	potential role of MMR/MSI anomalies as an indicator of an aggressive profile use of immune checkpoint inhibitors in ACC
potential applications of MMR/MSI in other endocrine tumors	yes: thyroid cancer and neuroendocrine neoplasia
Lynch syndrome (germline *MMR* mutation) and endocrine issues	➢ cancers belonging to gynecological endocrinology (endometrial, breast) ➢ challenges of pregnant females with Lynch syndrome ➢ early iatrogenic menopause-related issues ➢ ? ACC (not yet proven)
results: 10-year analysis ACC-MMR/MSI	11 studies
patients with Lynch syndrome and ACC	n = 2 N1 = 3 ACC /634 LS N2 = 2 ACC/135 LS
patients with ACC and MMR status	n = 3
	N1 = 364: 13.7% with non-germline MMR mutations N2 = 36: 8.57% with non-germline MMR mutations N3 = 94: 3.2% with germline MMR mutations
2 case series with Lynch syndrome	1 new case of ACC/ per paper
5 case reports	1 new case of ACC/per paper
first report of PD-1 blockade for ACC	in 2018

Abbreviations: ACC = adrenocortical carcinoma; MMR = mismatch repair; MSI = microsatellite instability; LS = Lynch syndrome.

## Data Availability

Not applicable.

## Abbreviations

ACC	adrenocortical carcinoma
APC	adenomatous polyposis coli
BC	breast cancer
CRC	colorectal cancer
EC	endometrial cancer
FNMTC	familial non-medullary thyroid cancer
HNPCC	hereditary non-polyposis colorectal cancer syndrome
IHC	immunohistochemistry
ICPI	immune checkpoint inhibitors
LS	Lynch syndrome
LOH	loss of heterozygosity
MMR	mismatch repair
MSI	microsatellite instability
MEN	multiple endocrine neoplasia
MiNEN	mixed neuroendocrine non-neuroendocrine neoplasm
NEN	neuroendocrine neoplasia
NGS	next-generation sequencing
OC	ovarian cancer
PCR	polymerase chain reaction
SNPs	single nucleotide polymorphisms
TC	thyroid cancer
WES	whole-exome sequencing

## References

[1] Wang Q. (2016). Cancer predisposition genes: Molecular mechanisms and clinical impact on personalized cancer care: Examples of Lynch and HBOC syndromes. Acta Pharmacol. Sin..

[2] Latham A., Srinivasan P., Kemel Y., Shia J., Bandlamudi C., Mandelker D., Middha S., Hechtman J., Zehir A., Dubard-Gault M. (2019). Microsatellite Instability Is Associated with the Presence of Lynch Syndrome Pan-Cancer. J. Clin. Oncol..

[3] Lynch H.T., Lynch P.M., Lanspa S.J., Snyder C.L., Lynch J.F., Boland C.R. (2009). Review of the Lynch syndrome: History, molecular genetics, screening, differential diagnosis, and medicolegal ramifications. Clin. Genet..

[4] Carethers J.M. (2015). Lynch syndrome and Lynch syndrome mimics: The growing complex landscape of hereditary colon cancer. World J. Gastroenterol..

[5] Li X., Liu G., Wu W. (2021). Recent advances in Lynch syndrome. Exp. Hematol. Oncol..

[6] Olave M.C., Graham R.P. (2021). Mismatch repair deficiency: The what, how and why it is important. Genes Chromosom. Cancer.

[7] Zhang L., Peng Y., Peng G. (2018). Mismatch repair-based stratification for immune checkpoint blockade therapy. Am. J. Cancer Res..

[8] Dominguez-Valentin M., Sampson J.R., Seppälä T.T., ten Broeke S.W., Plazzer J.-P., Nakken S., Engel C., Aretz S., Jenkins M.A., Sunde L. (2020). Cancer risks by gene, age, and gender in 6350 carriers of pathogenic mismatch repair variants: Findings from the Prospective Lynch Syndrome Database. Genet. Med..

[9] Lynch H.T., Shaw T.G. (2013). Practical genetics of colorectal cancer. Chin. Clin. Oncol..

[10] Tomita N., Ishida H., Tanakaya K., Yamaguchi T., Kumamoto K., Tanaka T., Hinoi T., Miyakura Y., Hasegawa H., Takayama T. (2021). Japanese Society for Cancer of the Colon and Rectum (JSCCR) guidelines 2020 for the Clinical Practice of Hereditary Colorectal Cancer. Int. J. Clin. Oncol..

[11] Wang C., Zhang L., Vakiani E., Shia J. (2022). Detecting mismatch repair deficiency in solid neoplasms: Immunohistochemistry, microsatellite instability, or both?. Mod. Pathol..

[12] Lalli E. (2021). ‘You cannot expect miracles to happen overnight’: Patience pays off when you wish to establish a new adrenocortical carcinoma cell line. Eur. J. Endocrinol..

[13] Jiang M., Jia K., Wang L., Li W., Chen B., Liu Y., Wang H., Zhao S., He Y., Zhou C. (2020). Alterations of DNA damage repair in cancer: From mechanisms to applications. Ann. Transl. Med..

[14] Biller L.H., Creedon S.A., Klehm M., Yurgelun M.B. (2021). Lynch Syndrome-Associated Cancers beyond Colorectal Cancer. Gastrointest. Endosc. Clin. N. Am..

[15] Da Costa W.H., Jabboure G., Da Cunha I.W. (2017). Urological cancer related to familial syndromes. Int. Braz. J. Urol..

[16] Vanoli A., Grillo F., Furlan D., Arpa G., Grami O., Guerini C., Riboni R., Mastracci L., Di Sabatino A. (2021). Small Bowel Epithelial Precursor Lesions: A Focus on Molecular Alterations. Int. J. Mol. Sci..

[17] Mete O., Erickson L.A., Juhlin C.C., de Krijger R.R., Sasano H., Volante M., Papotti M.G. (2022). Overview of the 2022 WHO Classification of Adrenal Cortical Tumors. Endocr. Pathol..

[18] Petr E.J., Else T. (2018). Adrenocortical carcinoma (ACC): When and why should we consider germline testing?. La Presse Médicale.

[19] Else T., Rodriguez-Galindo C. (2016). 5th International ACC Symposium: Hereditary Predisposition to Childhood ACC and the Associated Molecular Phenotype. Horm. Cancer.

[20] Kiseljak-Vassiliades K., Zhang Y., Bagby S.M., Kar A., Pozdeyev N., Xu M., Gowan K., Sharma V., Raeburn C.D., Albuja-Cruz M. (2018). Development of new preclinical models to advance adrenocortical carcinoma research. Endocr.-Relat. Cancer.

[21] Raygada M., Raffeld M., Bernstein A., Miettinen M., Glod J., Hughes M.S., Reilly K., Widemann B., Del Rivero J. (2021). Case report of adrenocortical carcinoma associated with double germline mutations in MSH2 and RET. Am. J. Med. Genet. Part A.

[22] Domènech M., Grau E., Solanes A., Izquierdo A., Del Valle J., Carrato C., Pineda M., Dueñas N., Pujol M., Lázaro C. (2021). Characteristics of Adrenocortical Carcinoma Associated with Lynch Syndrome. J. Clin. Endocrinol. Metab..

[23] Yang Z., Cheng H., Zhang Y., Zhou Y. (2021). Identification of NDRG Family Member 4 (NDRG4) and CDC28 Protein Kinase Regulatory Subunit 2 (CKS2) as Key Prognostic Genes in Adrenocortical Carcinoma by Transcriptomic Analysis. Experiment.

[24] Luo G., Chen G., Chen P., Zhou J. (2022). Pan-cancer analysis of histone methyltransferase KMT2D with potential implications for prognosis and immunotherapy in human cancer. Comb. Chem. High Throughput Screen..

[25] Raymond V.M., Everett J.N., Furtado L.V., Gustafson S.L., Jungbluth C.R., Gruber S.B., Hammer G.D., Stoffel E.M., Greenson J.K., Giordano T.J. (2013). Adrenocortical Carcinoma Is a Lynch Syndrome–Associated Cancer. J. Clin. Oncol..

[26] Brondani V.B., Montenegro L., Lacombe A.M.F., Magalhães B.M., Nishi M.Y., Funari M.F.d.A., Narcizo A.d.M., Cardoso L.C., Siqueira S.A.C., Zerbini M.C.N. (2020). High Prevalence of Alterations in DNA Mismatch Repair Genes of Lynch Syndrome in Pediatric Patients with Adrenocortical Tumors Carrying a Germline Mutation on *TP53*. Cancers.

[27] Jouinot A., Bertherat J. (2019). Diseases Predisposing to Adrenocortical Malignancy (Li–Fraumeni Syndrome, Beckwith–Wiedemann Syndrome, and Carney Complex). Genet. Endocr. Dis. Syndr..

[28] Challis B.G., Kandasamy N., Powlson A.S., Koulouri O., Annamalai A.K., Happerfield L., Marker A.J., Arends M.J., Nik-Zainal S., Gurnell M. (2016). Familial Adrenocortical Carcinoma in Association with Lynch Syndrome. J. Clin. Endocrinol. Metab..

[29] Kaur R.J., Pichurin P.N., Hines J.M., Singh R.J., Grebe S.K., Bancos I. (2019). Adrenal Cortical Carcinoma Associated with Lynch Syndrome: A Case Report and Review of Literature. J. Endocr. Soc..

[30] Wright J.P., Montgomery K.W., Tierney J., Gilbert J., Solórzano C.C., Idrees K. (2018). Ectopic, retroperitoneal adrenocortical carcinoma in the setting of Lynch syndrome. Fam. Cancer.

[31] Araújo A.N., Bugalho M.J. (2021). Advanced Adrenocortical Carcinoma: Current Perspectives on Medical Treatment. Horm. Metab. Res..

[32] Nevgi A., Klein O., Cheung A.S. (2020). Sustained remission of Lynch syndrome-associated metastatic adrenocortical carcinoma following checkpoint inhibitor therapy-associated multiorgan autoimmunity. Clin. Endocrinol..

[33] Ardolino L., Hansen A., Ackland S., Joshua A. (2020). Advanced Adrenocortical Carcinoma (ACC): A Review with Focus on Second-Line Therapies. Horm. Cancer.

[34] Casey R., Giger O., Seetho I., Marker A., Pitfield D., Boyle L., Gurnell M., Shaw A., Tischkowitz M., Maher E. (2018). Rapid disease progression in a patient with mismatch repair-deficient and cortisol secreting adrenocortical carcinoma treated with pembrolizumab. Semin. Oncol..

[35] Pozdeyev N., Fishbein L., Gay L.M., Sokol E.S., Hartmaier R., Ross J.S., Darabi S., Demeure M.J., Kar A., Foust L.J. (2021). Targeted genomic analysis of 364 adrenocortical carcinomas. Endocr.-Relat. Cancer.

[36] Cerquetti L., Bucci B., Carpinelli G., Lardo P., Proietti A., Saporito R., Rindi G., Petrangeli E., Toscano V., Stigliano A. (2019). Antineoplastic Effect of a Combined Mitotane Treatment/Ionizing Radiation in Adrenocortical Carcinoma: A Preclinical Study. Cancers.

[37] Aswath K., Welch J., Gubbi S., Veeraraghavan P., Avadhanula S., Gara S.K., Dikoglu E., Merino M., Raffeld M., Xi L. (2021). Co-Occurrence of Familial Non-Medullary Thyroid Cancer (FNMTC) and Hereditary Non-Polyposis Colorectal Cancer (HNPCC) Associated Tumors—A Cohort Study. Front. Endocrinol..

[38] Santos L.S., Silva S.N., Gil O.M., Ferreira T.C., Limbert E., Rueff J. (2018). Mismatch repair single nucleotide polymorphisms and thyroid cancer susceptibility. Oncol. Lett..

[39] Kim C.S., Mandel S.J. (2022). Contemporary Management of Thyroid Nodules. Annu. Rev. Med..

[40] Luo M., Huang Y., Li Y., Zhang Y. (2020). Metastatic rectal cancer to papillary thyroid carcinoma: A case report and review of literature. BMC Gastroenterol..

[41] Fazekas-Lavu M., Parker A., Spigelman A.D., Scott R.J., Epstein R.J., Jensen M., Samaras K. (2017). Thyroid cancer in a patient with Lynch syndrome—Case report and literature review. Ther. Clin. Risk Manag..

[42] Pozdeyev N., Rose M.M., Bowles D.W., Schweppe R. (2020). Molecular therapeutics for anaplastic thyroid cancer. Semin. Cancer Biol..

[43] Javid M., Sasanakietkul T., Nicolson N.G., Gibson C.E., Callender G.G., Korah R., Carling T. (2018). DNA Mismatch Repair Deficiency Promotes Genomic Instability in a Subset of Papillary Thyroid Cancers. World J. Surg..

[44] Fujita S., Masago K. (2021). Alteration of DNA mismatch repair capacity underlying the co-occurrence of non-small-cell lung cancer and nonmedullary thyroid cancer. Sci. Rep..

[45] Johnson J.M., Chen J., Ali S.M., Dardi I.K., Tuluc M., Cognetti D., Campling B., Sama A.R. (2018). Molecular Profiling of Synchronous Colon Cancers and Anaplastic Thyroid Cancer in a Patient with Lynch Syndrome. J. Gastrointest. Cancer.

[46] Genutis L.K., Tomsic J., Bundschuh R.A., Brock P.L., Williams M.D., Roychowdhury S., Reeser J.W., Frankel W.L., Alsomali M., Routbort M.J. (2019). Microsatellite Instability Occurs in a Subset of Follicular Thyroid Cancers. Thyroid.

[47] Rocha M.L., Schmid K.W., Czapiewski P. (2021). The prevalence of DNA microsatellite instability in anaplastic thyroid carcinoma—Systematic review and discussion of current therapeutic options. Contemp. Oncol./Współczesna Onkol..

[48] Wong K.S., Lorch J.H., Alexander E.K., Nehs M.A., Nowak J.A., Hornick J.L., Barletta J.A. (2019). Clinicopathologic Features of Mismatch Repair-Deficient Anaplastic Thyroid Carcinomas. Thyroid.

[49] Pelizzo M., Pennelli G., Zane M., Galuppini F., Colletti P., Boschin I.M., Rubello D. (2015). Papillary thyroid carcinoma (PTC) in Lynch syndrome: Report of two cases and discussion on Lynch syndrome behaviour and genetics. Biomed. Pharmacother..

[50] Romaniuk A., Lyndin M., Smiyanov V., Sikora V., Rieznik A., Kuzenko Y., Budko H., Moskalenko Y., Karpenko L., Gladchenko O. (2017). Primary multiple tumor with affection of the thyroid gland, uterus, urinary bladder, mammary gland and other organs. Pathol.-Res. Pract..

[51] Feng A.L., Le A., Johnson D.N., Varvares M.A. (2018). Multiple simultaneous head and neck cancers in Lynch syndrome: Case report and literature review. Laryngoscope.

[52] Pande M., Wei C., Chen J., Amos C.I., Lynch P.M., Lu K.H., Lucio L.A., Boyd-Rogers S.G., Bannon S.A., Mork M.E. (2012). Cancer spectrum in DNA mismatch repair gene mutation carriers: Results from a hospital based Lynch syndrome registry. Fam. Cancer.

[53] Caso R., Beamer M., Lofthus A.D., Sosin M. (2017). Integrating surgery and genetic testing for the modern surgeon. Ann. Transl. Med..

[54] Santos L.S., Gil O.M., Silva S.N., Gomes B.C., Ferreira T.C., Limbert E., Rueff J. (2020). Micronuclei Formation upon Radioiodine Therapy for Well-Differentiated Thyroid Cancer: The Influence of DNA Repair Genes Variants. Genes.

[55] Dumitru N., Ghemigian A., Carsote M., Albu S.E., Terzea D., Valea A. (2016). Thyroid nodules after initial evaluation by primary health care practitioners: An ultrasound pictorial essay. Arch. Balk. Med. Union..

[56] Verrienti A., Carbone A., Sponziello M., Pecce V., Cito D.S., Bruno R. (2022). Papillary thyroid carcinoma as first and isolated neoplastic disease in a Lynch syndrome family member with a germline MLH1 mutation. Endocrine.

[57] Qiao P.-P., Tian K.-S., Han L.-T., Ma B., Shen C.-K., Zhao R.-Y., Zhang Y., Wei W.-J., Chen X.-P. (2022). Correlation of mismatch repair deficiency with clinicopathological features and programmed death-ligand 1 expression in thyroid carcinoma. Endocrine.

[58] Pontoppidan K., Mathew R.P., Moran G.W. (2013). Acute dysphagia after a normal endoscopy: Think outside the box. Clin. Med..

[59] Mojtová E., Hanajíková J., Hamidová O., Bognár G., Dyttert D., Grigerová M., Kečkéš Š., Podoba J. (2020). An incidental finding of pheochromocytoma in a 33-year-old patient with Lynch syndrome. Vnitr. Lek..

[60] Riff B.P., Katona B., Wilkerson M., Nathanson K.L., Metz D.C. (2015). HNPCC-Associated Pheochromocytoma. Pancreas.

[61] Duraturo F., Liccardo R., De Rosa M., Izzo P. (2019). Genetics, diagnosis and treatment of Lynch syndrome: Old lessons and current challenges (Review). Oncol. Lett..

[62] Karimi M., Von Salomé J., Aravidis C., Silander G., Askmalm M.S., Henriksson I., Gebre-Medhin S., Frödin J.-E., Björck E., Lagerstedt-Robinson K. (2018). A retrospective study of extracolonic, non-endometrial cancer in Swedish Lynch syndrome families. Hered. Cancer Clin. Pract..

[63] Cox V.L., Bamashmos A.A.S., Foo W.C., Gupta S., Yedururi S., Garg N., Kang H.C. (2018). Lynch Syndrome: Genomics Update and Imaging Review. Radiographics.

[64] Farha N., Hrabe J., Sleiman J., Beard J., Lyu R., Bhatt A., Church J., Heald B., Liska D., Mankaney G. (2022). Clinically actionable findings on surveillance EGD in asymptomatic patients with Lynch syndrome. Gastrointest. Endosc..

[65] Kidambi T.D., Pedley C., Bergsland E.K., Terdiman J.P., Blanco A. (2017). Lower gastrointestinal neuroendocrine neoplasms associated with hereditary cancer syndromes: A case series. Fam. Cancer.

[66] Sekine R., Shimazu K., Nakano D., Yamaguchi T., Suzuki Y., Fukuda K., Yoshida T., Taguchi D., Iijima K., Nanjyo H. (2022). A novel Lynch syndrome pedigree bearing germ-line *MSH2* missense mutation c.1808A>T (Asp603Val). Jpn. J. Clin. Oncol..

[67] Sorscher S., Saroya B. (2013). A molecularly confirmed neuroendocrine tumor resulting from Lynch Syndrome. J. Gastrointest. Oncol..

[68] Lou L., Lv F., Wu X., Li Y., Zhang X. (2020). Clinical implications of mismatch repair deficiency screening in patients with mixed neuroendocrine non-neuroendocrine neoplasms (MiNEN). Eur. J. Surg. Oncol. (EJSO).

[69] Morani A.C., Hanafy A.K., Ramani N.S., Katabathina V.S., Yedururi S., Dasyam A.K., Prasad S.R. (2020). Hereditary and Sporadic Pancreatic Ductal Adenocarcinoma: Current Update on Genetics and Imaging. Radiol. Imaging Cancer.

[70] Grant R.C., Denroche R., Jang G.H., Nowak K.M., Zhang A., Borgida A., Holter S., Topham J.T., Wilson J., Dodd A. (2020). Clinical and genomic characterisation of mismatch repair deficient pancreatic adenocarcinoma. Gut.

[71] Aslanian H.R., Lee J.H., Canto M.I. (2020). AGA Clinical Practice Update on Pancreas Cancer Screening in High-Risk Individuals: Expert Review. Gastroenterology.

[72] Katabathina V.S., Buddha S., Rajebi H., Shah J.N., Morani A.C., Lubner M.G., Dasyam A., Nazarullah A., Menias C.O., Prasad S.R. (2021). Pancreas in Hereditary Syndromes: Cross-sectional Imaging Spectrum. RadioGraphics.

[73] Pittman M.E., Brosens L.A., Wood L.D. (2016). Genetic Syndromes with Pancreatic Manifestations. Surg. Pathol. Clin..

[74] Karamurzin Y., Zeng Z., Stadler Z.K., Zhang L., Ouansafi I., Al-Ahmadie H.A., Sempoux C., Saltz L.B., Soslow R.A., O’Reilly E.M. (2012). Unusual DNA mismatch repair–deficient tumors in Lynch syndrome: A report of new cases and review of the literature. Hum. Pathol..

[75] Barrera A.S., Pla S.S., Maña C.M.B., Salas R.C., Monforte N.G., González N.B., Monzonis A.R., Navarro F.J.A., Cueto M.R.B., Borobia F.G. (2017). Pancreatic non-functioning neuroendocrine tumor: A new entity genetically related to Lynch syndrome. J. Gastrointest. Oncol..

[76] Ban X., Mo S., Lu Z., Jia C., Shao H., Chang X., Mao X., Zhang Y., Pang J., Zhang Y. (2022). Expression and methylation status of MMR and MGMT in well-differentiated pancreatic neuroendocrine tumors and potential clinical applications. Endocrine.

[77] Koumarianou A., Kaltsas G.A., Chatzellis E., Kyriakopoulos G., Kolomodi D., Alexandraki K.I. (2021). Immunotherapeutics at the spearhead: Current status in targeting neuroendocrine neoplasms. Endocrine.

[78] Rekhi B., Menon S., Deodhar K.K., Ghosh J., Chopra S., Maheshwari A. (2020). Clinicopathological features of 50 mismatch repair (MMR)-deficient endometrial carcinomas, tested by immunohistochemistry: A single institutional feasibility study, India. Ann. Diagn. Pathol..

[79] Yousef I., Siyam F., Layfield L., Freter C., Sowers J.R. (2014). Cervical neuroendocrine tumor in a young female with Lynch Syndrome. Neuro Endocrinol. Lett..

[80] Shetty I., Fuller S., Raygada M., Merino M.J., Thomas B.J., Widemann B.C., Reilly K.M., Pacak K., Del Rivero J. (2020). Adrenocortical carcinoma masquerading as pheochromocytoma: A histopathologic dilemma. Endocrinol. Diabetes Metab. Case Rep..

[81] Calsina B., Piñeiro-Yáñez E., Martínez-Montes Á.M., Caleiras E., Fernández-Sanromán Á., Monteagudo M., Torres-Pérez R., Fustero-Torre C., Pulgarín-Alfaro M., Gil E. (2023). Genomic and immune landscape Of metastatic pheochromocytoma and paraganglioma. Nat. Commun..

[82] Inoue K., Kai K., Sato S., Nishida H., Hirakawa K., Nasu K., Narahara H. (2021). Mixed large and small cell neuroendocrine carcinoma and endometrioid carcinoma of the endometrium with high microsatellite instability: A case report and literature review. SAGE Open Med. Case Rep..

[83] Teodosescu A., Chan I., Elder J., Wu M. (2021). A correlation study of mismatch repair immunohistochemical protein expression of pancreatic solid tumors in cytology cell blocks and matching surgical specimens. Diagn. Cytopathol..

[84] Riccò B., Salati M., Bonetti L.R., Dominici M., Luppi G. (2020). PD-1 blockade in deficient mismatch repair mixed adenoneuroendocrine carcinoma of the stomach: New hope for an orphan disease. Tumori J..

[85] Luong T.V., Nisa Z., Watkins J., Hayes A.R. (2020). Should immunohistochemical expression of mismatch repair (MMR) proteins and microsatellite instability (MSI) analysis be routinely performed for poorly differentiated colorectal neuroendocrine carcinomas?. Endocrinol. Diabetes Metab. Case Rep..

[86] Sherman S.K., Schuitevoerder D., Chan C.H.F., Turaga K.K. (2020). Metastatic Colorectal Cancers with Mismatch Repair Deficiency Result in Worse Survival Regardless of Peritoneal Metastases. Ann. Surg. Oncol..

[87] Morgan S., Slodkowska E., Parra-Herran C., Mirkovic J. (2019). PD-L1,RB1 and mismatch repair protein immunohistochemical expression in neuroendocrine carcinoma, small cell type, of the uterine cervix. Histopathology.

[88] Liu I.H., Ford J.M., Kunz P.L. (2015). DNA-repair defects in pancreatic neuroendocrine tumors and potential clinical applications. Cancer Treat. Rev..

[89] Bengtsson D., Joost P., Aravidis C., Stenmark M.A., Backman A.-S., Melin B., Von Salomé J., Zagoras T., Gebre-Medhin S., Burman P. (2017). Corticotroph Pituitary Carcinoma in a Patient With Lynch Syndrome (LS) and Pituitary Tumors in a Nationwide LS Cohort. J. Clin. Endocrinol. Metab..

[90] Teuber J., Reinhardt A., Reuss D., Hähnel S., Unterberg A., Beynon C. (2021). Aggressive pituitary adenoma in the context of Lynch syndrome: A case report and literature review on this rare coincidence. Br. J. Neurosurg..

[91] Voisin M.R., Almeida J.P., Perez-Ordonez B., Zadeh G. (2019). Recurrent Undifferentiated Carcinoma of the Sella in a Patient with Lynch Syndrome. World Neurosurg..

[92] Uraki S., Ariyasu H., Doi A., Furuta H., Nishi M., Sugano K., Inoshita N., Nakao N., Yamada S., Akamizu T. (2017). Atypical pituitary adenoma with MEN1 somatic mutation associated with abnormalities of DNA mismatch repair genes; MLH1 germline mutation and MSH6 somatic mutation. Endocr. J..

[93] Loughrey P., Baker G., Herron B., Cooke S., Iacovazzo D., Lindsay J., Korbonits M. (2021). Invasive ACTH-producing pituitary gland neoplasm secondary to MSH2 mutation. Cancer Genet..

[94] Uraki S., Ariyasu H., Doi A., Kawai S., Takeshima K., Morita S., Fukai J., Fujita K., Furuta H., Nishi M. (2018). Reduced Expression of Mismatch Repair Genes MSH6/MSH2 Directly Promotes Pituitary Tumor Growth via the ATR–Chk1 Pathway. J. Clin. Endocrinol. Metab..

[95] Park D., Airi R., Sherman M. (2020). Microsatellite instability driven metastatic parathyroid carcinoma managed with the anti-PD1 immunotherapy, pembrolizumab. BMJ Case Rep..

[96] Andreasson A., Sulaiman L., Vale S.D., Martins J.M., Ferreira F., Miltenberger-Miltenyi G., Batista L., Haglund F., Björck E., Nilsson I.-L. (2012). Molecular characterization of parathyroid tumors from two patients with hereditary colorectal cancer syndromes. Fam. Cancer.

[97] Kostov S., Watrowski R., Kornovski Y., Dzhenkov D., Slavchev S., Ivanova Y., Yordanov A. (2022). Hereditary Gynecologic Cancer Syndromes—A Narrative Review. OncoTargets Ther..

[98] Aguirre E., Grana B., Boudet M., Balmaña J. (2016). Screening for Lynch Syndrome among Patients with Newly Diagnosed Endometrial Cancer: A Comprehensive Review. Tumori J..

[99] Zhao S., Chen L., Zang Y., Liu W., Liu S., Teng F., Xue F., Wang Y. (2022). Endometrial cancer in Lynch syndrome. Int. J. Cancer.

[100] Gallon R., Gawthorpe P., Phelps R.L., Hayes C., Borthwick G.M., Santibanez-Koref M., Jackson M.S., Burn J. (2021). How Should We Test for Lynch Syndrome? A Review of Current Guidelines and Future Strategies. Cancers.

[101] Corrado G., Marchetti C., Trozzi R., Scambia G., Fagotti A. (2021). Fertility preservation in patients with BRCA mutations or Lynch syndrome. Int. J. Gynecol. Cancer.

[102] Sheehan M., Heald B., Yanda C., Kelly E.D., Grobmyer S., Eng C., Kalady M., Pederson H. (2020). Investigating the Link between Lynch Syndrome and Breast Cancer. Eur. J. Breast Health.

[103] Sajjadi E., Venetis K., Piciotti R., Invernizzi M., Guerini-Rocco E., Haricharan S., Fusco N. (2021). Mismatch repair-deficient hormone receptor-positive breast cancers: Biology and pathological characterization. Cancer Cell Int..

[104] Brennan A., Brennan D., Rees M., Hickey M. (2021). Management of menopausal symptoms and ovarian function preservation in women with gynecological cancer. Int. J. Gynecol. Cancer.

[105] Fedda F.A., Euscher E.D., Ramalingam P., Malpica A. (2020). Prophylactic Risk-reducing Hysterectomies and Bilateral Salpingo-oophorectomies in Patients With Lynch Syndrome: A Clinicopathologic Study of 29 Cases and Review of the Literature. Int. J. Gynecol. Pathol..

[106] Etchegary H., Dicks E., Tamutis L., Dawson L. (2018). Quality of life following prophylactic gynecological surgery: Experiences of female Lynch mutation carriers. Fam. Cancer.

[107] Holter S., Hall M.J., Hampel H., Jasperson K., Kupfer S.S., Haidle J.L., Mork M.E., Palaniapppan S., Senter L., Stoffel E.M. (2022). Risk assessment and genetic counseling for Lynch syndrome—Practice resource of the National Society of Genetic Counselors and the Collaborative Group of the Americas on Inherited Gastrointestinal Cancer. J. Genet. Couns..

[108] Stupart D., Win A.K., Winship I.M., Jenkins M. (2015). Fertility after young-onset colorectal cancer: A study of subjects with Lynch syndrome. Color. Dis..

[109] Terribas E., Bonache S., García-Arévalo M., Sánchez J., Franco E., Bassas L., Larriba S. (2010). Changes in the Expression Profile of the Meiosis-Involved Mismatch Repair Genes in Impaired Human Spermatogenesis. J. Androl..

[110] Al-Obaidy K.I., Trevino K.E., Idrees M.T. (2019). Clinicopathologic Characterization of Bilateral Testicular Germ Cell Tumors with Immunohistochemical Evaluation of Mismatch Repair and BRAF (V600E) Genes Mutations. Int. J. Surg. Pathol..

[111] Rudolph C., Melau C., Nielsen J.E., Jensen K.V., Liu D., Pena-Diaz J., Meyts E.R.-D., Rasmussen L.J., Jørgensen A. (2017). Involvement of the DNA mismatch repair system in cisplatin sensitivity of testicular germ cell tumours. Cell. Oncol..

[112] Jimenez C., Armaiz-Pena G., Dahia P.L.M., Lu Y., Toledo R.A., Varghese J., Habra M.A. (2022). Endocrine and Neuroendocrine Tumors Special Issue-Checkpoint Inhibitors for Adrenocortical Carcinoma and Metastatic Pheochromocytoma and Paraganglioma: Do They Work?. Cancers.

[113] Petris R., Valea A., Rentea D.E., Gheorghe A.M., Ghemigian A., Carsote M., Petrova E., Sandru F., Haldan A., Nistor C.E. (2022). Pitfalls of adrenal tumors’ management in real-life medicine: A cases series. Manag. Health.

[114] Habra M.A., Stephen B., Campbell M., Hess K., Tapia C., Xu M., Rodon Ahnert J., Jimenez C., Lee J.E., Perrier N.D. (2019). Phase II clinical trial of pembrolizumab efficacy and safety in advanced adrenocortical carcinoma. J. Immunother. Cancer.

[115] Shen C., Wang Y. (2023). Ferroptosis Biomarkers for Predicting Prognosis and Immunotherapy Efficacy in Adrenocortical Carcinoma. Arch. Med. Res..

[116] Guan Y., Yue S., Chen Y., Pan Y., An L., Du H., Liang C. (2022). Molecular Cluster Mining of Adrenocortical Carcinoma via Multi-Omics Data Analysis Aids Precise Clinical Therapy. Cells.

[117] Ławnicka H. (2023). Current Prospects for Adrenocortical Carcinoma Pharmacotherapy. Recent Patents Anti-Cancer Drug Discov..

[118] Bates M.F., Sorensen M.J. (2023). Genetic Testing for Adrenal Tumors—What the Contemporary Surgeon Should Know. Surg. Oncol. Clin. N. Am..

[119] Riedmeier M., Thompson L.D.R., Molina C.A.F., Decarolis B., Härtel C., Schlegel P.-G., Fassnacht M., Wiegering V. (2023). Prognostic value of the Weiss and Wieneke (AFIP) scoring systems in pediatric ACC—A mini review. Endocrine-Related Cancer.

[120] Sneha L., Saminathan T., Dhivyalakshmi J., Joseph L. (2022). Precocious puberty in a child: A rare cause and review of literature. J. Fam. Med. Prim. Care.

[121] Brönimann S., Garstka N., Remzi M. (2023). Treatment of adrenocortical carcinoma: Oncological and endocrine outcomes. Curr. Opin. Urol..

